# Development of a pragmatic and brief wellbeing tool for public health promotion: the mental wellbeing indicator (MWI)

**DOI:** 10.3389/fpsyg.2025.1627029

**Published:** 2026-01-05

**Authors:** Emily Brindal, Timothy F. Bainbridge, Naomi Kakoschke

**Affiliations:** Commonwealth Scientific Industrial Research Organisation, Health and Biosecurity, Human Health, Adelaide, SA, Australia

**Keywords:** psychological wellbeing, outcome assessment, health promotion, health behavior, vitality

## Abstract

**Background:**

Despite growing emphasis on mental wellbeing as a critical part of health, few tools allow the public to track and understand it. Our aim was to develop a brief wellbeing tool for public health promotion and research that could be offered directly to the public to assist in improving community wellbeing literacy.

**Methods:**

The study involved the completion of a single-time, anonymous online survey administered to panel members of an independent market research company. Eligible participants were aged 18 years and over, residing in Australia and not self-identifying as experiencing considerable struggles with emotions or stress. Measures included existing tools theoretically aligned with existing wellbeing indexes (Satisfaction with Life, Psychological Wellbeing Scale, Mental-Health Consortium) and scales capturing theorized elements of state and trait wellbeing across several theoretical conceptions (e.g., positive affect, hope, self-efficacy, self-esteem, social support).

**Results:**

The final sample included 1,267 adults (50.4% male, 32.8% aged 25–44 years) which was split into a training sample (*n* = 887) for factor identification and item selection, and a test sample for confirmation (*n* = 380). A series of exploratory and confirmatory factor analyses identified three core constructs (Subjective Wellbeing, Perceived Social Support and Authenticity). The final 11-item set fit the data well (i.e., Comparative Fit Index > 0.99). Overall, the three constructs were moderately related to each other and aligned with existing wellbeing theories. Together these were named the Mental Wellbeing Indicator (MWI). Correlations with other indicators including mental health diagnosis and resilience suggested convergent and divergent validity. Predictive validity was demonstrated strongly for Subjective Wellbeing, moderately for Perceived Social Support and weakly for Authenticity.

**Conclusion:**

These findings largely demonstrate the validity and utility of the tool for assessing wellbeing and predicting important outcomes. After further study, the MWI could become a promising wellbeing tool in public health promotion.

## Introduction

Wellbeing is widely recognized as a critical domain of general health (World Health Organisation, 2022). Yet, interpretations vary, ranging from those which are all encompassing including aspects of physical, emotional, financial, social, and mental wellbeing to those much more narrowly focused on the emotional and cognitive evaluations (i.e., happiness and life satisfaction) (Park et al., 2023). Despite multiple measures and theories, there is no single, accepted encompassing approach to wellbeing which creates confusion in the community and makes health promotion activities difficult. The purpose of the current study was to assess different areas of wellbeing and understand how they may cluster and relate to one another with the purpose of engaging the public in health promotion regarding wellbeing. Advancing our understanding and conceptualization of wellbeing is important not only from a scientific perspective, but also to improve public health monitoring and messaging.

### Leading theoretical frameworks of wellbeing

Theoretical models provide conceptual guidance on the key components of wellbeing. Lambert et al. (2015), used 27 theories to illustrate different wellbeing theory and concepts. Often wellbeing is separated by philosophical concepts of *hedonia*, seeking positive feelings or happiness, and *eudaimonia*, seeking purpose (Lambert et al., 2015). Although philosophically different, dichotomizing the conceptualization of wellbeing may not be as useful in practice with appraisals of meaning in life bridging the theoretical gap (Steger, 2016).

Most existing measures are based on the two popular models of wellbeing (Linton et al., 2016), namely, subjective wellbeing (Diener, 1984) and psychological wellbeing (Ryff and Keyes, 1995). Within the definition of subjective wellbeing, Diener (1984) discusses three core elements: virtue, life satisfaction and happiness. Three key characteristics associated with subjective wellbeing include: presence of a person’s own evaluation (subjective), focus on the experience of positives rather than the absence of negatives and global scale (covering most aspects of a person’s life). This early conception of wellbeing strongly aligns strongly with satisfaction with life, which is captured in most wellbeing studies (van Agteren et al., 2021) and combines leading philosophies of *hedonia* and *eudaimonia*. Ryff (1989) proposed a model of wellbeing that extended beyond happiness to more strongly emphasize the role of meaning and what she labels psychological wellbeing. Ryff and Keyes (1995) introduced a six-factor model that measured self-acceptance, environmental mastery, positive relations (with others), purpose in life, personal growth, and autonomy which when combined, comprise “psychological wellbeing.” Innovative parts of psychological wellbeing theory include the inclusion of a life course perspective demonstrating how these elements shift at different life stages, and recognition that wellbeing can remain high in the absence of pleasurable feelings such as happiness. While the Psychological Wellbeing Scale was developed to measure these six domains, studies have failed to confirm the presence of six distinct factors in the associated scale (Burns and Machin, 2009).

Although not a specific theory, Positive Psychology has added a variety of concepts and philosophy to the wellbeing space including flourishing, hope, and, vitality and the pursuit of happiness (Seligman and Csikszentmihalyi, 2000; Seligman, 2002) which is also core to Diener’s components of subjective wellbeing. One highly cited theory from positive psychology suggests that positive affective states can lead to upward spiraling, essentially the opposite to downward spiraling (Fredrickson, 2001). Extending concepts from subjective wellbeing, a virtuous cycle has also been suggested, where virtue creates happiness and the alternative (Kesebir and Diener, 2014). Literature in this field has led to the development of concepts such psychological capital, which details how hope, efficacy, resilience and optimism can be used to leverage greater wellbeing or experience of happiness with the fundamental idea that those with higher positive psychological resources can better navigate inevitable life challenges (Luthans et al., 2014). The Mental Health Continuum (MHC) (Keyes, 2006) is frequently used as a measure of wellbeing aligned broadly with Positive Psychology theory and is designed to capture flourishing and languishing. Recently, it has been shown to have a three factor structure capturing emotional, social and psychological wellbeing and been described as measuring “general mental wellbeing” (Iasiello et al., 2022).

More recently, efforts have been made to summarize “emotional wellbeing.” A proposed framework encompasses a mixture of subjective, mental and psychological wellbeing with the aim of describing emotional wellbeing and reconciling existing theories (Park et al., 2023). The framework offers 21 terms considered central to the definition of emotional wellbeing such as “affect wellbeing,” “happiness,” “life satisfaction” and “meaning.”

There is no one accepted theoretical definition of wellbeing with suggestion of subjective, psychological, mental, emotional wellbeing and many others. Yet outside of specific theoretical or philosophical allegiance, there are commonalities in the psychological exploration of wellbeing. Purpose, life satisfaction and meaning are shared across all conceptions. There is philosophical divide between the importance of positive feelings which are emphasized in most conceptions except psychological wellbeing which instead focusses on purposeful pursuits.

#### Existing wellbeing indexes and measures for public health

There is a need to understand and monitor wellbeing in combination with physical and mental health as a key but distinct indicator of general health. To do this effectively, it is essential to capture measurable elements of wellbeing critical to determining health outcomes, and to do so with brief measures appropriate for the broader population.

All states and territories in the Australia, published wellbeing frameworks in 2022, but only four included specific indicator metrics (Act Government, 2025; Australian Government Productivity Commission, 2025; Australian Government The Treasury, 2023; SA Health, 2025). Some of these metrics were aligned with poor mental health rather than indicators for intervention. For example, Closing the Gap (Australian Government Productivity Commission, 2025) which is targeted at improving health of indigenous people, includes only suicide rates as indicators of wellbeing. Suicide remains difficult to predict statistically (Belsher et al., 2019) and is only moderately associated with life satisfaction and happiness (Bray and Gunnell, 2006). More recently, the Australian government introduced the Measuring What Matters framework to assess population-level wellbeing based on broad elements of health, mental health, productivity, cohesion and mental wellbeing which is assessed only through Life Satisfaction (Australian Bureau of Statistics, 2024).

Two decades ago, a critical review of wellbeing noted the diversity of definition that has been associated with wellbeing in research and practice, and that while this increases its general appeal, it can also lead to misinterpretation and cross-purpose (de Chavez et al., 2005). A good example of this in the health field is the use of quality-of-life assessment. These tools are designed to inform care practice with a focus on patient reporting within the context of medical treatments or chronic health conditions (Addington-Hall and Kalra, 2001). They capture elements of both functional capacity (i.e., ability to move and complete daily tasks) and broader patient wellbeing including emotional and social wellbeing generally in the context of a condition (Cella, 1994). General tools have also been developed to included happiness (Costanza et al., 2007).

The ability of wellbeing constructs to be predictive of health outcomes outside of medical treatment and specific conditions is a key indicator of appropriateness for use in public health. A meta-analysis exploring subjective wellbeing and health across experimental and longitudinal studies differentiated wellbeing measures based on affective states (i.e., mood, hope) and trait characteristics (i.e., life satisfaction, happiness, optimism) (Howell et al., 2007). Results suggested that state-based measures have a strong association with short-term outcomes (e.g., pain tolerance and immune function), while trait measures are associated with longer-term outcomes such as cardiovascular disease. Thus, both could contribute to overall health via different pathways. The advantage of considering state-based elements is that there are multiple offerings to improve mood, happiness, and satisfaction (Ghielen et al., 2018; Schutte and Malouff, 2019). Unfortunately, many of the analyses of health and wellbeing in epidemiology and medicine have relied on secondary data where variables are retrospectively combined to represent wellbeing (Hernandez et al., 2018).

Even fewer peer-reviewed wellbeing tools exist which are designed specifically to allow an individual to understand and track important aspects of their own mental wellbeing. In the United Kingdom, the BBC developed a consumer-facing subjective wellbeing scale that covers physical health, relationships and psychological wellbeing (Kinderman et al., 2011; Pontin et al., 2013). It was completed by more than 23,000 members of the public as part of The Stress Test conducted with BBC Lab UK. The original tool captured four domains of quality of life, six of psychological wellbeing, and used three items to assess thoughts on self, world and future that are generally associated with low mood (Kinderman et al., 2011). The psychological wellbeing questions covered perceptions of self, autonomy, growth, achievement, purpose, enjoyment and depression. The final subscales showed moderate correlation to the Goldberg Anxiety and Depression scales, indicators of poor mental health, but were not compared to validated wellbeing scales. Inclusion of depression as a wellbeing outcome again blurs the distinction between wellbeing and mental health, which although associated, are likely to be distinct with theorists noting a distinction between objective health and subjective wellbeing outcomes (Lomas et al., 2024).

### The current research

Despite the growing emphasis and interest in wellbeing, few validated tools exist that allow people to track and understand their own wellbeing. The multiple conceptions of wellbeing in psychology only further confusion; as do public facing indicators or tools which emphasize limited objective data (i.e., suicide rates) (Australian Government Productivity Commission, 2025) and blur the distinction between mental health and wellbeing (Kinderman et al., 2011) which may explain why community members struggle to differentiate mental health and wellbeing (Chng et al., 2022). Diverse descriptions of wellbeing are frequently loyal to key theories which while philosophically different, may not be comprehended this way by the public. Finally, despite frequent inclusion in public health monitoring, and strong theoretical basis, satisfaction with life is a global, high-level indicator offering little deeper insight for a person completing it. Despite a seemingly intuitive comprehension of this concept, how it is formed remains the subject of academic exploration (Rojas and Elizondo-Lara, 2012). It is difficult to directly influence as it is likely to be the end point resulting from other wellbeing indicators and consist of several life domains which vary by number, type and intensity individually.

Our primary aim was to develop a brief wellbeing index for public health promotion that was true to well-accepted theories of wellbeing and valid as an indicator of mental wellbeing. The wider objective was to create a measure that was both academically sound to enable valid monitoring, but also appropriate for health promotion—thus having the ability to provide feedback on more than one domain and preferably capturing domains with high comprehensibility enabling it to be offered directly to the public as a tool to assist them in understanding individual wellbeing with the longer-term ambition of improving community wellbeing literacy. In doing this, a secondary aim of the current study was to examine how various proposed conceptual elements of wellbeing group together outside of assessments developed closely with specific theories. Therefore, a variety of measures capturing shared elements of subjective wellbeing as defined by Diener (1984), psychological wellbeing as defined by Ryff and Keyes (1995) and positive psychology constructs were included measuring both state and trait level constructs aligned with broader health outcomes. Specific measures were included if they focussed on wellbeing constructs and the presence of positive characteristics, rather than the absence of negative ones (Diener, 1984). They also needed to be focussed on wellbeing rather than mental health. This meant the experience of depressive and anxiety symptoms, and negative affect were not included.

To capture state-based indicators, mood, vitality and hope were included. Mood and hope can predict health outcomes (Howell et al., 2007). Vitality is widely included in Positive Psychology research and has been associated with weight loss and the experience of physical symptoms (Ryan and Frederick, 1997). More recently, there has also been recognition of vitality as an indicator of psychological wellbeing in medicine (Rozanski, 2023). The final state-based wellbeing domains considered in the current study were purpose and authenticity (Kernis and Goldman, 2006) which align with psychological wellbeing theory. Authenticity has not been strongly featured in existing theory but has ties to autonomy and meaning and is of growing interest (Lutz et al., 2023; Wilt et al., 2021). It has been argued that self-realization is a significant component of eudaimonia and that this is reflected in performing activities that reflect one’s calling and therefore greater authenticity (Kernis and Goldman, 2006).

Trait-based indicators included self-efficacy and self-esteem. Self-efficacy has been strongly associated with a variety of health behaviors (Schwarzer and Jerusalem, 1995) and, links strongly to evaluations of the self which is proposed by psychological wellbeing theory. Lastly, self-esteem is a fundamental part of self, heavily underlying self-evaluation and acceptance (Brown et al., 2001), and has links with both mental and physical health (Mann et al., 2004; Zell and Johansson, 2025).

In addition to state and trait measures, we included social support and positive evaluations of relations with others. Diener and Seligman (2002) indicate that good social relationships are “necessary for happiness.” Social appraisals (e.g., “Society if becoming a better place for people”) and belonging are captured in the MHC (Keyes, 2006). In addition to appraisals of society, perceived social support has been shown to be predictive of total life satisfaction across ages (Siedlecki et al., 2014) and has been considered part of wellbeing in population assessment as well as by theorists (Diener and Seligman, 2002; Turner, 1981).

## Materials and methods

### Participants

The aim was to recruit 1,000–1,500 people aged 18 years and over currently residing in Australia. Exclusion criterion included anyone self-identifying as currently experiencing considerable struggles with emotions or stress primarily for ethical reasons—to minimize any distress caused to participants given the nature of the wellbeing questions that ask people to reflect about their life and context.

### Procedure

The study involved the completion of a single-time, anonymous online survey that was administered by an independent national market research company to its panel members. The company emailed existing members with an invitation and interested participants were directed to a link displaying a participant information sheet and consent box. Those consenting then completed the 15-min survey online. Data were collected over 2 weeks in July 2023. Participants received loyalty rewards within the market research system upon completion of the survey. The study was approved by CSIRO’s Low-Risk Human Ethics Committee (2023_017_LR).

#### Measures

Scales were chosen to capture the identified constructs of interest based on evidence of previous validation, no licensing requirements to facilitate public accessibility, and broad application in the literature. Items were presented on a standard 7-point agreement scale unless otherwise described.

#### Existing wellbeing measures

##### Life satisfaction

Diener et al.’s (1985) original five item Satisfaction with Life Scale was used with total scores calculated based on the sum of all items.

##### Psychological wellbeing

This was measured through the 42-item psychological wellbeing scale (Ryff, 1989). The ratings scale was presented on a standard 7-point agreement scale rather than the original 6-point version due to a programing error.

##### Mental health continuum

The short form of the MHC (Keyes, 2006) was included to capture a global measure of mental wellbeing aligned with positive psychology. Items are rated according to how often they are experienced: Never, once or twice, about once a week, about 2–3 times a week, almost everyday.

##### Resilience

To further assess convergent validity, resilience was included. Resilience describes the ability to bounce back after adversity (Smith et al., 2008). It has been considered an important recurring theme in the study of wellbeing (Ryff, 2014). It has been related to healthy aging at the population level (Cosco et al., 2017). The 6 items from the Brief Resilience Scale (Smith et al., 2008) were presented on a 5-point agreement scale and then summed to represent higher resilience.

#### State measures

##### Mood

The Activation and Safe/Content Affect Scale (ASCAS) was used to assess affect. It measures three subscales of positive emotions – activation, safety and contentment (Gilbert et al., 2008). Mood items are rated from 0 (not characteristic of me) to 4 (very characteristic of me).

##### Hope

The 6-item State Hope Scale (Snyder et al., 1996) was used in its published form to capture two aspects of hope (agency and pathways). Items are rated on an 8-point scale from false to true.

##### Vitality

The original 7-item scale (Bostic et al., 2000) was used as instructed to capture vitality including a single reversed item.

##### Authenticity

The 45-item authenticity scale (Kernis and Goldman, 2006) was used to capture four sub-domains of authenticity (awareness, unbiased processing, behavior, and relational orientation). Items were all rated on a standard 5-point agreement scale.

##### Purpose

The 6-item Life Engagement Test (Scheier et al., 2006) was used to measure levels purpose. Items were rated on a 5-point agreement scale.

#### Trait measures

##### Self-efficacy

Ten items from the Generalized Self-Efficacy scale (Schwarzer and Jerusalem, 1995) were presented on a 4-point scale according to how true the participant believed each statement to be as a reflection of them.

##### Self-esteem

Rosenberg 10-item self-esteem scale was presented on a 4-point agreement scale. Items were reverse-coded when needed and summed to represent higher self-esteem (Rosenberg, 1965).

#### Social measures

##### Social support

The 12-item Multidimensional Scale of Perceived Social Support (MSPSS) (Zimet et al., 1988) was used to capture social support. Items are summed to create total scores for three subscales capturing the support of significant other, family and friends.

##### All-humanity

The all-humanity scale (McFarland et al., 2012) was used to capture a sense of broader empathy and evaluation of society. It was presented in its original form with nine questions presented three times according to different categories “People in my community,” “Australians” and “People all over the world.” Responses could be made on a 5-point scale.

#### Demographic variables

Participants were asked which gender they identified with (male, female, non-binary, prefer not to say), their age group in 5-year brackets and other demographic characteristics. They were asked to report on the presence any mental health diagnoses in the last 12 months, their disability status (Madden et al., 2020), the presence of chronic health conditions, experience of significant life events (selected from pre-specified eight events) and to rate their overall health using self-rated health responses (Poor, Fair, Good, Very Good, Excellent).

### Data analysis

The market research company screened all data based on implausible response times, removing 40 cases prior to providing a final dataset of 1,501 responses. These data were further cleaned based on extreme mean responding on balanced scales (i.e., scales with negatively worded items) before reverse scoring, and straight-lining (long strings of the same values). Specifically, mean responses < 1.5 or > 3.5 on self-esteem (on a 1–4 scale) or mean responses < 2 or > 4 on resilience (1–5 scale) were excluded and anyone with a long string > 30 were excluded. The long string length was chosen conservatively based on a histogram of maximum long string lengths. This resulted in the removal of 234 cases and an analytical dataset with 1,267 participants (Table 1). The sample was randomly split into training (70%; *n* = 887) and test (30%; *n* = 380) subsamples with the training set used for item selection and the test set used for scale evaluation. Samples were combined for assessment of predictive utility.

**TABLE 1:** Participant demographics for final analytical data set (*n* = 1,267).

Variable	*n*	%
**Gender**		
Females	622	49.1
Males	638	50.4
Non-binary/not reported	7	<1%
**Age (years)**		
18–24	123	9.7
25–34	208	16.4
35–44	208	16.4
45–54	200	15.8
55–64	206	16.3
65–74	175	13.8
75 or older	147	11.6
**Self-rated health**		
Excellent	84	6.6
Very Good	301	23.8
Good	523	41.3
Fair	287	22.7
Poor	72	5.7
**Number of life events selected**		
None	703	55.5
1	356	28.1
2	137	10.8
3 or more	71	5.6
Mental health diagnosis (yes)	364	28.7
**Disability status**		
None	665	52.5
Experience difficulty but use technology to assist	169	13.3
Experience difficulty	433	34.2
**Number of chronic health conditions selected**		
None	866	68.4
1	154	12.2
2	80	6.3
3 or more	167	13.2

#### Training set, item selection (*n* = 887)

Each existing scale was evaluated in a confirmatory factor analysis (CFA). Scales that failed to pass a lenient fit (Comparative Fit Index value, CFI > 0.85) were not included in the EFA and item selection process.

Exploratory factor analysis (EFA) was performed at an item level including each of the remaining scales. Parallel analysis based on a reduced correlation matrix (Humphreys and Montanelli, 1975) was used to estimate the maximum number of factors to extract. EFAs were conducted with Minimum Residual Method and Oblimin rotations. Solutions were evaluated based on parsimony and how well the solutions accommodated the items (based on complexity and communality).

Factors of the selected solution were evaluated in bifactor models, with the factors forming the general factors and items from each scale forming the group factors. Maximum Likelihood method was used. Modification index values were examined and adjustments made for models with CFI < 0.95. Initial item sets were selected for each factor based on the highest loading items on the general factor from each scale.

To further refine the items, configural, metric, and scalar measurement invariance for age group, bachelor’s degree status (yes/no), and gender (male/female) were examined using multigroup CFA (Maassen et al., 2025). Scales passed configural invariance assessment if the CFI was >0.95, and metric and scalar invariance if change in CFI was equal or > –0.01 (ΔCFI ≥ –0.01) compared to the preceding model. As the models were nested (i.e., each model was a restricted version of the prior model), failure to pass a particular measurement invariance test at one level implied failure at all subsequently levels. When scalar invariance was not achieved, partial invariance was used to discover which items were responsible. The worst item was then replaced by performing the same measurement invariance test with each of the alternative items from the same original scale. The replacement item was selected from those that resulted in the scale achieving scalar measurement invariance (if any such item existed) based on the highest general factor loading, or the item that came closest to achieving scalar measurement invariance (based on ΔCFI) if none of the iterations passed the test. This procedure was repeated for each of the categories considered (i.e., age, bachelor’s degree status, and gender) to arrive at the final item sets.

#### Test set, scale validation (*n* = 380)

Identified item sets were tested in CFAs within the test set to check they had appropriate measurement models. Acceptable fit was evaluated as CFI >0.95. The scales were then tested for measurement invariance over the same categories as used in item selection in the training sample.

Finally, the validity of each facet and the total scale were assessed. For convergent validity, factor scores were calculated by scaling the items such that their minimum was 1 and maximum was 5 to account for items with different measurement scales. Mean scores of the items were used to compute correlations with Psychological Wellbeing, the MHC sub scales and Satisfaction with Life Scores. To test divergent validity, resulting scores were correlated with mental health diagnosis and resilience.

#### Predictive validity (*n* = 1,267)

The entire sample was recombined for analyses exploring predictive utility to allow optimal Power to detect smaller effects. Predictive validity was tested with self-rated health and satisfaction with life as outcomes, and gender, age, mental health diagnosis, number of health conditions and significant life events, and resilience as the covariates. To avoid inflated Type 1 error rates latent variable models were used (Westfall and Yarkoni, 2016), and to avoid interpretational confounding, measurement parameters were fixed in the structural models (Burt, 1976). A baseline model regressed self-rated health on the covariates and allowed the target variable to correlate with the residual variance of self-rated health (Hayes, 2021). A second model then added the target variable as a predictor and the change in the variance explained between the models (Δ*R*^2^) was computed. Bootstrap confidence intervals for ΔR^2^ were calculated by a function, using 1,000 bootstrap samples (Hayes, 2021).

## Results

Participant characteristics in terms of age and gender were broadly representative of the Australian population (Table 1). All scales showed excellent internal consistency based on Cronbach Alpha scores (Table 2), but confirmatory factor analysis of led to the exclusion of the Unbiased Processing facet of Authenticity and the All-Humanity Scale from further analysis (Comparative Fit Index value, CFI < 0.85) (Table 3).

**TABLE 2:** Scale summary statistics for all measures included in the current study (*n* = 1,267).

Scale	Items	α	M	SD
**Existing measures**				
Satisfaction with life	5	0.917	22.19	7.17
Mental health continuum	14	0.943	39.95	15.83
Psychological wellbeing	42	0.944	139.19	36.82
**State-based measures**				
ASCAS: Activation	8	0.912	17.99	6.89
ASCAS: Relaxed	6	0.897	14.82	5.33
ASCAS: Safe	4	0.851	10.68	3.54
Hope: Pathways	3	0.900	15.61	4.63
Hope: Agency	3	0.897	14.60	5.16
Vitality	7	0.910	4.14	1.35
Authenticity: Awareness	12	0.843	42.79	6.96
Authenticity: Relational orientation	12	0.777	42.92	6.08
Authenticity: Behavioral	11	0.754	36.65	5.88
Life engagement test	6	0.879	22.06	4.96
**Trait-based measures**				
Generalized self-efficacy	10	0.919	29.37	5.51
Resilience	6	0.874	16.80	4.50
Self-esteem	10	0.917	28.90	6.41
**Social measures**				
MSPSS: Significant other	4	0.961	20.21	7.00
MSPSS: Family	4	0.921	19.17	6.18
MSPSS: Friends	4	0.923	18.76	5.85

All-Humanity and Authenticity (Unbiased Processing) scales were removed prior to analysis due to poor fit indices within the training sample (Comparative Fit Index value, CFI > 0.85). ASCAS, Activation and Safe/Content Affect Scale; MSPSS, Multidimensional Scale of Perceived Social Support.

**TABLE 3:** Fit indices for scales in included in study (training sample only; *n* = 887).

Scale	χ^2^	*df*	*p*	CFI	RMSEA	95% CI
ASCAS: Activation	211.69	20	< 0.001	0.95	0.10	[0.09, 0.12]
ASCAS: Relaxed	79.39	9	< 0.001	0.98	0.09	[0.08, 0.11]
ASCAS: Safe	62.60	2	< 0.001	0.96	0.18	[0.15, 0.23]
Vitality	49.97	14	< 0.001	0.99	0.05	[0.04, 0.07]
MSPSS: Significant other	3.54	2	0.170	1.0	0.03	[0.00, 0.08]
MSPSS: Family	66.83	2	< 0.001	0.98	0.19	[0.15, 0.23]
MSPSS: Friends	46.81	2	< 0.001	0.99	0.16	[0.12, 0.20]
All-humanity	710.03	27	< 0.001	0.81	0.17	[0.16, 0.18]
Self-esteem	169.17	30	< 0.001	0.97	0.07	[0.06, 0.08]
Life Engagement test	45.64	6	< 0.001	0.98	0.09	[0.06, 0.11]
Authenticity: Awareness	278.35	50	< 0.001	0.93	0.07	[0.06, 0.08]
Authenticity: Behavioral	246.66	38	< 0.001	0.89	0.08	[0.07, 0.09]
Authenticity: Relational orientation	294.05	51	< 0.001	0.90	0.07	[0.07, 0.08]
Authenticity: Unbiased processing	379.89	35	< 0.001	0.77	0.11	[0.10, 0.12]
Generalized self-efficacy	230.07	35	< 0.001	0.96	0.08	[0.07, 0.09]
Hope: Pathways	0.00	0		1.0	0.00	[0.00, 0.00]
Hope: Agency	0.00	0		1.0	0.00	[0.00, 0.00]

ASCAS, Activation and Safe/Content Affect Scale; MSPSS, Multidimensional Scale of Perceived Social Support.

### Factor analysis and item selection in training set (*n* = 887)

Kaiser-Meyer-Olkin Index value was 0.97 and Bartlett’s test was significant [χ^2^(5,356) = 64,635, *p* < 0.001], indicating the sample was appropriate for EFA. Parallel analysis suggested up to 11 factors. The 11-factor solution looked reasonable apart from some moderately large factor correlations (i.e., 0.60), and separate facets from single scales loading on their own factors. Selecting fewer factors, down to 4, mostly looked reasonable with facets from scales progressively combining or closely related scales occupying the same factor. At 3 factors, some of the items were poorly described and there were many cross-loadings with the positive and reversed authenticity items largely failing to occupy the same factor. Given that negatively worded items from other scales loaded with their associated construct, we concluded that the negatively worded authenticity items may measure a different construct from authenticity. We excluded the negatively worded authenticity items on the basis that the positively worded items were more closely associated to the original construct and performed a new set of EFAs.

Parallel analysis of the reduced set of items suggested that up to 10 factors were reasonable (Supplementary material). Results were broadly similar to those of the first set of EFAs with the omission of a factor for the reversed authenticity items. Given our preference for parsimony, the 3-factor solution was selected and is presented in Table 4.

**TABLE 4:** Factor extraction results based on Minimum Residual Method and Oblimin rotations in the test sample (*n* = 887).

Item	Factor 1	Factor 2	Factor 3	Complexity	Communality
V7-I feel energized	0.80			1.1	0.62
V4-I have energy and spirit	0.79			1.1	0.63
**V1-I feel alive and vital**	0.78			1.1	0.66
GSE7-I can remain calm when facing difficulties because I can rely on my coping abilities	0.71			1.2	0.46
ACS1-Energetic	0.71			1.1	0.43
GSE4-I am confident that I could deal efficiently with unexpected events	0.69			1.2	0.46
ACS2-Lively	0.69			1.0	0.45
V6-I nearly always feel awake and alert	0.69			1.0	0.47
**ACS16-Content**	0.67			1.3	0.58
ACS4-Active	0.66			1.1	0.38
V5-I look forward to each new day	0.66			1.2	0.6
GSE5-Thanks to my resourcefulness, I know how to handle unforeseen situations.	0.64			1.3	0.41
**HOPE4-Right now I see myself as being pretty successful.**	0.64			1.1	0.56
**GSE3-It is easy for me to stick to my aims and accomplish my goals.**	0.64			1.1	0.42
ACS6-Dynamic	0.64			1.0	0.34
ACS17-Secure	0.64			1.1	0.53
ACS8-Eager	0.63			1.0	0.39
GSE9-If I am in trouble, I can usually think of a solution	0.63			1.3	0.43
HOPE6-At this time, I am meeting the goals that I have set for myself.	0.63			1.1	0.57
HOPE5-I can think of many ways to reach my current goals.	0.62			1.2	0.57
ACS9-Relaxed	0.62			1.1	0.40
GSE8-When I am confronted with a problem, I can usually find several solutions.	0.62			1.2	0.40
ACS5-Enthusiastic	0.62			1.1	0.49
ACS14-Serene	0.61			1.1	0.39
HOPE2-At the present time, I am energetically pursuing my goals.	0.61			1.1	0.51
GSE10-I can usually handle whatever comes my way.	0.60			1.3	0.42
V3-Sometimes I am so alive I just want to burst	0.59			1.3	0.33
HOPE3-There are lots of ways around any problem that I am facing now.	0.59			1.2	0.50
ACS7-Excited	0.59			1.2	0.35
HOPE1-If I should find myself in a jam, I could think of many ways to get out of it.	0.58			1.4	0.55
SE10-I take a positive attitude toward myself.	0.58			1.3	0.54
ACS12-Tranquil	0.58			1.0	0.32
SE6-I certainly feel useless at times.	–0.58			1.1	0.43
ACS3-Adventurous	0.56			1.1	0.24
GSE6-I can solve most problems if I invest the necessary effort.	0.56			1.1	0.40
ACS11-Calm	0.56			1.4	0.37
GSE1-I can always manage to solve difficult problems if I try hard enough	0.56			1.6	0.39
ACS10-Peaceful	0.55			1.1	0.34
ACS15-Safe	0.52			1.2	0.42
**SE1-On the whole, I am satisfied with myself.**	0.51			1.6	0.54
SE2-At times I think I am no good at all.	–0.50			1.3	0.42
MDPSS2-There is a special person with whom I can share joys and sorrows.		0.82		1.0	0.68
**MDPSS1 -There is a special person who is around when I am in need.**		0.82		1.0	0.68
MDPSS5-I have a special person who is a real source of comfort to me.		0.81		1.0	0.65
MDPSS10-There is a special person in my life who cares about my feelings.		0.79		1.0	0.66
**MDPSS4-I get the emotional help & support I need from my family.**		0.75		1.0	0.62
MDPSS8-I can talk about my problems with my family.		0.71		1.1	0.60
MDPSS11-My family is willing to help me make decisions.		0.71		1.0	0.55
MDPSS3-My family really tries to help me.		0.70		1.0	0.51
**MDPSS9-I have friends with whom I can share my joys and sorrows.**		0.68		1.1	0.57
MDPSS6-My friends really try to help me.		0.66		1.0	0.46
MDPSS12-I can talk about my problems with my friends.		0.65		1.1	0.55
MDPSS7-I can count on my friends when things go wrong.		0.64		1.1	0.55
AUTH29-I actively attempt to understand myself as best as possible.			0.60	1.0	0.42
**AUTH43-The people I am close to can count on me being who I am regardless of what setting we are in.**			0.58	1.1	0.40
AUTH9-I have a very good understanding of why I do the things I do.			0.58	1.3	0.49
AUTH20-I am aware of when I am not being my true-self.			0.57	1.2	0.27
AUTH25-I try to act in a manner that is consistent with my personally held values, even if others criticize or reject me for doing so.			0.57	1.0	0.35
AUTH44-My openness and honesty in close relationships are extremely important to me.			0.56	1.2	0.36
AUTH3-For better or for worse I am aware of who I truly am.			0.56	1.2	0.42
AUTH4-I understand why I believe the things I do about myself.			0.55	1.1	0.37
AUTH5-I want people with whom I am close to understand my strengths.			0.53	1.2	0.28
AUTH23-It is important for me to understand my close others’ needs and desires.			0.53	1.5	0.34
AUTH21-I am able to distinguish those self-aspects that are important to my core-or true-self from those that are unimportant.			0.53	1.3	0.39
**AUTH28-I find that my behavior typically expresses my values.**			0.53	1.2	0.43
AUTH24-I want close others to understand the real me rather than just my public persona or “image.”			0.50	1.5	0.25
**AUTH38-I am in touch with my motives and desires.**			0.50	1.7	0.49
AUTH40-In general, I place a good deal of importance on people I am close to understanding who I truly am.			0.50	1.3	0.31

Final items selected for abbreviated measure are bolded. Factor correlations F1->F2, r = 0.49; F1->F3, r = 0.46; F2->F3, r = 0.35. It is suggested that items be presented on a 5-point agreement scale in future administration.

Factor 1 consisted of items from vitality, self-esteem, self-efficacy and hope scales, capturing a mixture of state and trait-based measures crossing theoretical wellbeing domains of energy, self-acceptance and hope. This factor was labeled “Subjective Wellbeing.” Factor 2 included all the social support items and therefore was labeled “Perceived Social Support” in line with the original scale. Factor 3 included 15 of the items from the authenticity scale. These largely represented the awareness (7/12 original items) and relational orientation (6/12 original items) subscales which capture awareness of motives, feelings and thoughts and the ability to achieve genuine relationships with others. It was labeled “Authenticity.”

Most items from nearly all scales had their primary loading on the same factor as other items from the scale. Exceptions included Life Engagement, where all items had moderate loadings on at least 2 of the 3 factors, and a single authenticity behavior item loaded primarily on a different factor to all other items from the same scale. As a result, life engagement was excluded from the item selection process.

To help refine the factors, separate bifactor models were estimated for each of the 3 factors with a general factor and a group factor for each constituent scales. All models had adequate fit (i.e., CFI > 0.90), given their complexity, but modification indices for Subjective Wellbeing suggested that the two hope facets should not be treated separately (i.e., the expected parameter change for allowing the facets to correlate was 0.98). A subsequent model combined these into a single group factor, which also had adequate fit.

Items were subsequently selected for each scale based on the highest loading item on the general factor for each scale. Of these initial item sets, F1 and F3 failed to achieve scalar invariance by age and F2 failed to achieve scalar invariance by bachelor’s degree status. Partial invariance tests identified the items most responsible for the lack of invariance and these items were replaced with the item from the same scale that passed the measurement invariance test or failed by the smallest margin. The final set of items are highlighted in Table 4.

### Validation and validity in test set (*n* = 380)

Factor 1 (Subjective Wellbeing) had strong internal consistency [Cronbach alpha 0.810; *M* = 3.44 (SD = 0.83)] as did Factor 2 (Perceived Social Support) [Cronbach alpha 0.781; *M* = 3.61(SD = 0.97)]. The final factor (Authenticity) had acceptable consistency [Cronbach alpha 0.634; *M* = 3.83 (SD = 0.62)].

Subjective Wellbeing was tested in a CFA. Perceived Social Support and Authenticity were not tested in CFAs as they were only just identified, but they were tested in multigroup CFAs for the measurement invariance tests (next paragraph) and achieved adequate fit there (see the Supplementary material). The Subjective Wellbeing CFA fit well, χ^2^(5) = 12.1, *p* = 0.033, CFI = 0.991, RMSEA = 0.061 (90% CI:0.016, 0.106), SRMR = 0.021.

Measurement invariance across gender, age, and bachelor’s degree status are summarized in Table 5. Subjective Wellbeing and Perceived Social Support were scalar invariant across all tests, suggesting that means and correlations can be validly compared across gender, age, and bachelor’s degree status for these scales (Borsboom, 2006). Authenticity was metric invariant by age and bachelor’s degree status, but only configural invariant by gender. Therefore, when using the Authenticity scale, group means should not be compared and correlations should not be compared between genders.

**TABLE 5:** Summary of measurement invariance results in the test sample (*n* = 887).

Characteristic	Subjective wellbeing	Perceived social support	Authenticity
Gender (male/female)	Scalar	Scalar	Configural
Age group	Scalar	Scalar	Metric
Bachelor’s degree (yes/no)	Scalar	Scalar	Metric

Full statistics available in Supplementary material.

Correlations with Satisfaction with Life, Resilience, MHC facets, Psychological Wellbeing, and mental health diagnosis are reported in Table 6. Moderate to large correlations with measures closely related to wellbeing (i.e., Psychological Wellbeing, Satisfaction with Life, Resilience, and MHC facets) suggested convergent validity and small to moderate correlations with Mental Health, and number of conditions and life events suggested divergent validity. Larger correlations for Subjective Wellbeing compared to the other two scales, suggests it is the most important of the three factors for both wellbeing and good mental health.

**TABLE 6:** Bivariate correlation values to assess convergent and divergent validity in test sample (*n* = 380).

Variable	Subjective wellbeing	Perceived social support	Authenticity
Psychological wellbeing	0.797	0.560	0.614
Satisfaction with life	0.766	0.553	0.419
MHC—hedonic	0.773	0.527	0.450
MHC—social	0.581	0.297	0.342
MHC—psychological	0.721	0.487	0.510
Resilience	0.539	0.246	0.336
Mental health diagnosis (yes)	–0.306	–0.057	–0.123
Number of chronic health conditions (0–3 or more)	–0.388	–0.181	–0.103
Number of significant life events (0–3 or more)	–0.197	–0.098	0.047

### Predictive validity (*n* = 1,267)

Predictive validity analysis results predicting self-rated health are reported in Tables 7, 8. Subjective Wellbeing almost tripled the R^2^ of Satisfaction with Life and contributed to a moderate ΔR^2^ for self-reported health. Perceived Social Support contributed moderately to the prediction of satisfaction with life but only minimally to self-reported health. Authenticity contributed to a small ΔR^2^ for satisfaction with life and minimally to self-reported health.

**TABLE 7:** Predictive validity of subjective wellbeing, perceived social support and authenticity for self-rated health (*n* = 1,267).

Variable	B	SE	Beta	*Z*-value	*P*
**Subjective wellbeing [*R*^2^ = 0.391; Δ*R*^2^ = 0.162 (0.123, 0.206)]**					
Gender (male)	–0.045	0.048	–0.022	–0.94	0.350
Age group	–0.121	0.013	–0.225	–9.00	< 0.001
Mental health diagnosis (yes)	–0.071	0.057	–0.033	–1.25	0.210
Number of chronic health conditions (0–3 or more)	–0.177	0.024	–0.189	–7.32	< 0.001
Number of significant life events (0–3 or more)	–0.048	0.027	–0.042	–1.77	0.077
Resilience	–0.055	0.033	–0.055	–1.69	0.092
Subjective wellbeing	0.536	0.035	0.536	15.33	< 0.001
**Perceived social support [*R*^2^ = 0.271; Δ*R*^2^ = 0.041 (0.012, 0.063)]**					
Gender (male)	–0.024	0.052	–0.012	–0.46	0.646
Age group	–0.120	0.014	–0.223	–8.29	< 0.001
Mental health diagnosis (yes)	–0.192	0.060	–0.090	–3.22	0.001
Number of chronic health conditions (0–3 or more)	–0.252	0.025	–0.270	–10.02	< 0.001
Number of significant life events (0–3 or more)	–0.071	0.029	–0.063	–2.46	0.014
Resilience	0.162	0.029	0.162	5.61	< 0.001
Perceived social support	0.216	0.029	0.217	7.37	< 0.001
**Authenticity [*R*^2^ = 0.264; Δ*R*^2^ = 0.034 (0.013, 0.063)]**					
Gender (male)	–0.035	0.052	–0.017	–0.66	0.508
Age group	–0.129	0.015	–0.240	–8.51	< 0.001
Mental health diagnosis (yes)	–0.184	0.060	–0.086	–3.04	0.002
Number of chronic health conditions (0–3 or more)	–0.257	0.025	–0.276	–10.14	< 0.001
Number of significant life events (0–3 or more)	–0.084	0.029	–0.074	–2.88	0.004
Resilience	0.139	0.031	0.139	4.44	< 0.001
Authenticity	0.211	0.036	0.211	5.89	< 0.001

Dependent variable = self-rated health. Reduced model *R*^2^ = 0.230.

**TABLE 8:** Predictive validity of subjective wellbeing, perceived social support and authenticity for satisfaction with life (*n* = 1,267).

Variable	B	SE	Beta	*Z*-value	*P*
**Subjective wellbeing [*R*^2^ = 0.720; Δ*R*^2^ = 0.468 (0.414, 0.522)]**					
Gender (male)	0.032	0.030	0.016	1.07	0.284
Age group	–0.008	0.010	–0.014	–0.72	0.471
Mental health diagnosis (yes)	0.033	0.046	0.015	0.70	0.482
Number of chronic health conditions (0–3 or more)	0.035	0.021	0.037	1.67	0.094
Number of significant life events (0–3 or more)	–0.087	0.023	–0.076	–3.78	< 0.001
Resilience	–0.110	0.028	–0.110	–3.93	< 0.001
Subjective wellbeing	0.913	0.030	0.913	30.44	< 0.001
**Perceived social support [*R*^2^ = 0.480; Δ*R*^2^ = 0.231 (0.184, 0.281)]**					
Gender (male)	0.144	0.036	0.072	4.01	< 0.001
Age group	–0.013	0.012	–0.024	–1.04	0.296
Mental health diagnosis (yes)	–0.139	0.054	–0.065	–2.58	0.010
Number of chronic health conditions (0–3 or more)	–0.084	0.024	–0.090	–3.55	< 0.001
Number of significant life events (0–3 or more)	–0.119	0.027	–0.104	–4.43	< 0.001
Resilience	0.225	0.027	0.225	8.27	< 0.001
Perceived social support	0.515	0.027	0.515	18.80	< 0.001
**Authenticity [*R*^2^ = 0.338; Δ*R*^2^ = 0.089 (0.053, 0.135)]**					
Gender (male)	0.128	0.040	0.064	3.19	0.001
Age group	–0.005	0.014	–0.009	–0.33	0.738
Mental health diagnosis (yes)	–0.124	0.059	–0.058	–2.08	0.037
Number of chronic health conditions (0–3 or more)	–0.102	0.026	–0.109	–3.95	< 0.001
Number of significant life events (0–3 or more)	–0.136	0.029	–0.119	–4.62	< 0.001
Resilience	0.224	0.032	0.224	7.00	< 0.001
Authenticity	0.344	0.037	0.344	9.42	< 0.001

Dependent variable = Satisfaction with Life. Reduced model *R*^2^ = 0.249.

## Discussion

The current study aimed to create a short and robust wellbeing tool for the Australian population for public health monitoring and to improve public health promotion and communication. Results based on this Australian sample (*n* = 1,267) suggested a three-factor structure of wellbeing consisting of Subjective Wellbeing, Perceived Social Support and Authenticity. Reduction of scales items into a shorter form was successful with the 11-item solution generated in the training sample confirmed in the test sample. Correlations with other indicators and mental health suggested convergent and divergent validity. Predictive validity was demonstrated strongly for Subjective Wellbeing and moderately for Perceived Social Support but less so for Authenticity. These findings largely demonstrate the validity and utility of the tool for assessing wellbeing and predicting important outcomes. Overall, the three constructs extracted are well aligned with aspects of all existing wellbeing theories and were moderately related to each other as would be expected. Given this, we have labeled these items together, the Mental Wellbeing Indicator (MWI).

The first factor, namely, “Subjective Wellbeing,” refers largely to emotional and self-evaluative components of wellbeing with energy elements contributing strongly. The label Subjective Wellbeing aligns with broader criteria for subjective wellbeing in that these are all global, personal and positive reflections (Diener, 1984). It deviates from the cognitive judgments of virtue and purpose originally aligned to this label and emphasizes components of self-evaluation and acceptance that are more typically aligned with the Psychological Wellbeing Scale and the MHC.

Previously, theories have preferred to separate emotional and self-evaluative concepts of wellbeing. This may be in part due to philosophical divide between the pursuit of happiness (*hendonia*) versus purpose (*eudaimonia*) (Lambert et al., 2015). In line with philosophical division, the recent “emotional wellbeing” framework notes concepts such as “ability to pursue goals” and “acceptance” as peripheral (Park et al., 2023). The current analysis suggests that self-evaluative components previously associated with eudaimonia can fit with hedonic wellbeing. Interestingly, others have also suggested that concepts such vitality (which loaded strongly onto this factor) cross philosophical distinction (Hernandez et al., 2018). Many of the self-evaluative components used in this survey do appear to reflect feelings about oneself, so their grouping with affective evaluations has face validity. It also suggests that Subjective Wellbeing in this instance could be described as feelings expressed through mood, as well as feelings created by evaluations of oneself and abilities.

Based on its performance as a measure, Subjective Wellbeing was the strongest predictive indicator of life satisfaction as well as self-rated health. It includes both state- and trait-based elements which present different possible avenues for impacts on health (Ghielen et al., 2018; Howell et al., 2007). State-based elements such as mood provide excellent for possible intervention (Ghielen et al., 2018). A strength of this tool is that it considered both high intensity positive affect (i.e., vitality) as well as lower intensity ones (i.e., contentment) which may have appeal to different dispositional preferences (Hills and Argyle, 2001). Trait variables such as self-esteem may be more difficult to change in the short-term but also have multiple avenues of influence that could be subject of intervention and education.

At face value, the idea that a person who is happy with themselves, believes in their abilities and feels high levels of positive energy rings true to Positive Psychology concepts such as upward spiraling and psychological capital. Previous reviews suggest that self-esteem could be an indicator of better self-regulatory strategies and more adaptive goal pursuit as well as happiness (Baumeister et al., 2003). Subjective wellbeing was moderately associated with resilience which also provides some support for the idea that these factors represent “doing better” across a variety of domains (Freire and Ferreira, 2020), and that resilience and wellbeing are somehow related (Ryff, 2014).

The second distinct factor identified was Perceived Social Support based on a shortened version of the original scale. This factor had predictive utility for life satisfaction but minimal value to self-rated health. The role of social support in general health has been well-established, although the mechanisms are poorly understood (Schwarzer and Leppin, 1991). It may be that social support influences both the way we behave in relation to maintaining health but also impacts psychological processes and outcomes (Uchino, 2006). The inclusion of social support as a core construct in an assessment of overall wellbeing is well supported. Our specific measurement of social support failed to capture loneliness, which has recently attracted attention for its associations with mortality and physical health issues (Hawkley, 2022) but which also focusses on the negative aspects of experience rather than the positive. However, one review suggested that social support is moderately negatively related to loneliness *(r* = –0.39) (Zhang and Dong, 2022), suggesting that perceived social support likely captures some of the negative effects of loneliness while also capturing the positive effects of social support. The scale we included to capture evaluations of broader belonging failed to meet generous fit indicators which means we were unable to consider the more evaluative components of social life. It was interesting that this factor associated more strongly with psychological and emotional aspects of wellbeing than the social ones captured in MHC.

The final factor captured items reflecting authenticity, mostly centered on greater self-awareness and genuine relationships with other people. The concept of authenticity crosses philosophical, psychological and moral bounds. It has been philosophized that authenticity could be considered a virtue (i.e., being fully human), but the items here more closely resemble the idea of being true to oneself (Guignon, 2008). Kernis and Goldman (2006) associate authenticity with the pursuit of eudaimonia based on the idea that being authentic is about pursuing one’s true potential. Through this lens, it makes sense that this is loaded as a construct separate to the self-evaluations in the Subjective Wellbeing factor. These items do not reflect how well you believe you are doing, but about how well you know yourself and are living your values. This could align with purpose considered by many theories to be a central part of wellbeing and would also account for the observed cross-loadings Life Engagement Test items designed to capture purpose.

Despite the relevance of authenticity for wellbeing, there are several words of caution. Kernis and Goldman (2006) presented a four-factor conceptualization in their measure and one of these factors did not have good fit in the current sample. Given the relative novelty of authenticity as a wellbeing indicator, further work is needed to measure its validity. Also, authenticity provided minimal utility to the prediction of self-reported health and only modestly to satisfaction with life. It may be that this construct is important for wellbeing but has less immediate effects on health and wellbeing—as has been explored in a growing body of momentary work (Lutz et al., 2023).

### Limitations and future work

The scale was developed entirely from a single data source, so should be confirmed in a novel sample to ensure generalizability of the 11 items, and the underlying resonance of items in the community. We will need further work to assess this, and stability over time.

The focus of this study has been the Australian context due to its aim to offer a public facing tool in an Australian domain. There is no underlying reason to believe that this is particularly unique to other cultures with similar societal structure. There have been notable differences in self-evaluative measures between cultures that differ in individualistic versus collective mindsets (Diener and Diener, 1995) which means that significant cultural differences are likely to interfere with the performance of Subjective Wellbeing, as any other tool featuring components of self-evaluation. We excluded people self-identified levels of high distress which also reduces generalizability to the broader population. As the final aim for this tool was to provide feedback about wellbeing and not mental health, to support psychological safety, use of this tool will also aim to discourage those in current high states of distress who will be redirected to more diagnostically appropriate avenues.

The composition of Subjective Wellbeing observed in this study could be indicative of the broader construct of “happiness.” We did not measure general happiness which means we cannot understand the potential relationship between the two, or the incremental validity of this factor against general happiness. A study of over 900 Portuguese adolescents suggested that self-esteem and life satisfaction were moderators between negative states (depression and stress) and happiness (Freire and Ferreira, 2020) which supports the notion that it could be separate but key to the pursuit of *hedonia*. Nonetheless, further work is needed to validate this tool against happiness as well as other indicators of poor mental health such as distress.

Finally, the results are based on the variables entered. It is possible that a different assessment of purpose for example, may have loaded differently. However, all scales were chosen based on some level of validation that they assess the constructs of interest.

The three factors identified provide direction for a consumer-facing wellbeing tool that can show people how different aspects of their feelings, self-evaluation, authenticity and relationships with others may impact their psychological wellbeing, and possibly their health. These factors are clearly identifiable and provide excellent utility in the prediction of life satisfaction which is a preferred population indicator of wellbeing. The difference is that each of these factors also provide tangible avenue for self-reflection and change and therefore are ideal targets for health promotion. Sparse existing work has sought to promote mental wellbeing to the public (Canning et al., 2023) potentially due to confusion and controversy in the definition of wellbeing generated in the literature.

In the future we plan to incorporate new methods to provide users with a more personal experience. For example, while theoretical debate continues as to the importance of psychological, emotional, mental and general wellbeing, few have asked the community what is central to them. A recent measure of wellbeing asked subjects to rate 11 life domains associated with subjective wellbeing and used best-worse scaling to differentiate importance as well as satisfaction (Burke et al., 2024). The authors report that personal relationships are both highly important and provide high life satisfaction while good health is essential but provides limited feelings of satisfaction. Our future research aims to employ similar methods to understand the perceived value of each of the constructs we have identified for the public, moving toward greater utility for engagement, improving wellbeing literacy and informing health promotion efforts.

## Conclusion

Wellbeing is an essential but distinct part of health, with increasing recognition (World Health Organisation, 2022). Theoretical models have been essential to inform psychological investigations and to describe and capture various types of wellbeing. However, theories can also lead to insular thinking and circular investigations. Positive Psychology has attracted strong criticism in this regard (Kristjánsson, 2010). Competing theoretical approaches can also lead to increasingly academic debate, and result in outcomes further separated from everyday context. It was our aim to develop a simple tool informed by (but not wedded to) wellbeing theory to begin the conversation with the public about wellbeing and facilitate greater health promotion efforts and, over time, improve community engagement and literacy. We have presented findings which support the development of a new, brief pragmatic tool for capturing and intervening in mental wellbeing in this context. This tool captures levels of subjective wellbeing, relationships with others and authenticity and is predictive of self-rated health and satisfaction with life. Future revision will ensure its appropriateness for the public, confirm the validity of its structure and assess its ability to improve wellbeing literacy.

## Data Availability

The raw data supporting the conclusions of this article are not readily available and are subject to ethical approval. Requests to access the datasets should be directed to the corresponding author.
