# Supplementary material for: Development of a pragmatic and brief wellbeing tool for public health promotion: the mental wellbeing indicator (MWI)
Source: Front Psychol. 2026 Jan 5;16:1627029. doi: 10.3389/fpsyg.2025.1627029 (PMC12813144; doi:10.3389/fpsyg.2025.1627029)

Supplementary Materials Table S1. Measurement Invariance statistics for the final scales in the test data

Grouping	Type	χ^2	*df*	*p*	AIC	CFI	$\Delta\chi^2$	Δ*df*	*p*	ΔCFI
					Subjective wellbeing					
Gender	Configural	18.1	10	.053	5,104	0.99				
	Metric	24.8	14	.036	5,103	0.99	6.7	4	.153	-.003
	Scalar	31.2	18	.027	5,101	0.98	6.4	4	.171	-.003
	Strict	36.3	23	.039	5,096	0.98	5.0	5	.413	.000
Age Group	Configural	27.8	15	.023	5,113	0.98				
	Metric	38.4	23	.023	5,107	0.98	10.6	8	.223	-.003
	Scalar	51.2	31	.013	5,104	0.97	12.8	8	.118	-.006
	Strict	75.0	41	<.001	5,108	0.96	23.8	10	.008	-.018
Bachelor's Degree	Configural	16.8	10	.078	5,062	0.99				
	Metric	21.0	14	.102	5,058	0.99	4.1	4	.387	.000
	Scalar	28.4	18	.057	5,057	0.99	7.4	4	.117	-.004
	Strict	32.6	23	.088	5,051	0.99	4.3	5	.510	.001
					Perceived Social Support					
Gender	Configural	0.0	0		4,150	1.00				
	Metric	4.0	2	.133	4,150	0.99	4.0	2	.133	-.006
	Scalar	6.6	4	.160	4,149	0.99	2.5	2	.282	-.001
	Strict	7.6	7	.365	4,144	1.00	1.1	3	.783	.005
Age Group	Configural	0.0	0		4,157	1.00				
	Metric	0.8	4	.938	4,150	1.00	0.8	4	.938	.000
	Scalar	4.2	8	.842	4,145	1.00	3.4	4	.499	.000
	Strict	24.4	14	.041	4,153	0.97	20.2	6	.003	-.029
Bachelor's Degree	Configural	0.0	0		4,116	1.00				
	Metric	0.7	2	.708	4,113	1.00	0.7	2	.708	.000
	Scalar	1.7	4	.782	4,110	1.00	1.1	2	.589	.000
	Strict	4.7	7	.698	4,107	1.00	2.9	3	.401	.000

Grouping	Type	χ^2	*df*	*p*	AIC	CFI	$\Delta\chi^2$	Δ*df*	*p*	ΔCFI
					Authenticity					
Gender	Configural	0.0	0		2,637	1.00				
	Metric	7.0	2	.030	2,640	0.96	7.0	2	.030	-.036
	Scalar	7.4	4	.117	2,637	0.98	0.3	2	.841	.012
	Strict	10.4	7	.169	2,634	0.98	3.0	3	.395	.000
Age Group	Configural	0.0	0		2,611	1.00				
	Metric	0.9	4	.925	2,604	1.00	0.9	4	.925	.000
	Scalar	11.6	8	.170	2,607	0.97	10.7	4	.030	-.028
	Strict	42.2	14	<.001	2,626	0.78	30.6	6	<.001	-.189
Bachelor's Degree	Configural	0.0	0		2,626	1.00				
	Metric	1.0	2	.603	2,623	1.00	1.0	2	.603	.000
	Scalar	6.1	4	.189	2,625	0.98	5.1	2	.077	-.016
	Strict	7.1	7	.417	2,620	1.00	1.0	3	.807	.015

Supplementary Figure 1: Scree plot based on parallel analysis of all items in the training sample (n=887)

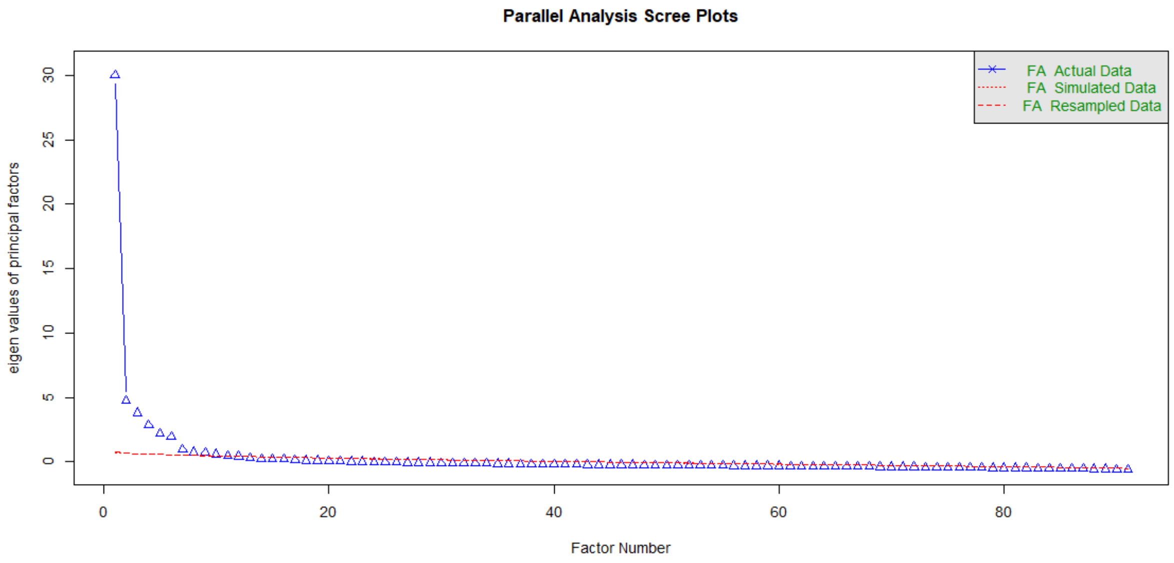

Supplement: Supplementary file 1 [file Data_Sheet_1.pdf]
